# Potential of *Beauveria bassiana* in the control of *Euschistus crenator* (Hemiptera: Pentatomidae) and selectivity to the parasitoid *Telenomus podisi* (Hymenoptera: Scelionidae)

**DOI:** 10.1002/ps.70614

**Published:** 2026-02-03

**Authors:** Paulo Henrique Martins da Silva, Gustavo Andrade Carneiro, Ricardo Antonio Polanczyk

**Affiliations:** ^1^ Plant Protection Department, Faculty of Agrarian and Veterinary Sciences Universidade Estadual Paulista “Júlio de Mesquita Filho” São Paulo Brazil; ^2^ Microbial Control of Pests Laboratory (LCMAP), Faculty of Agrarian and Veterinary Sciences Universidade Estadual Paulista “Júlio de Mesquita Filho” São Paulo Brazil

**Keywords:** biological control, entomopathogenic fungi (EPFs), intraguild interaction, natural enemy, stink bugs

## Abstract

**BACKGROUND:**

The combination of entomopathogenic fungi (EPFs) with natural enemies represents a promising strategy for more sustainable management programs within the context of integrated pest management (IPM). This study aimed to evaluate the pathogenicity of EPF isolates on 2^nd^‐instar nymphs of *Euschistus crenator*, through daily mortality and estimation of lethal time, in addition to investigating the selectivity of the most efficient isolates with respect to the egg parasitoid *Telenomus podisi*, an important natural enemy of stink bugs in soybean crops.

**RESULTS:**

All isolates were pathogenic to *E. crenator*, with LCMAP106, UFSM‐01, and the commercial strain PL63 (BOV) of *Beauveria bassiana* promoting 83.75%, 75% and 87.5% mortality, respectively, after 10 days. The biological parameters of the offspring of females of *T. podisi* exposed to these isolates were evaluated. The exposure did not affect parasitism or survival, although it did influence the sex ratio, emergence and egg‐to‐adult cycle. This study also confirmed, for the first time, the parasitism of *E. crenator* eggs by *T. podisi*.

**CONCLUSION:**

The results demonstrate the potential of different fungal isolates in controlling *E. crenator* and indicate that *T. podisi* can be used in conjunction with EPFs, as its ability to parasitize pest eggs and interrupt their biological cycle was not affected. © 2026 The Author(s). *Pest Management Science* published by John Wiley & Sons Ltd on behalf of Society of Chemical Industry.

## Graphical abstract

Entomopathogenic fungi effectively suppressed the emerging soybean pest *Euschistus crenator*, with isolates LCMAP106, UFSM‐01 and a commercial bioinsecticide achieving the highest mortality. These agents did not reduce parasitism or survival of the egg parasitoid *Telenomus podisi*, highlighting their potential for integration into environmentally sustainable pest management programs.

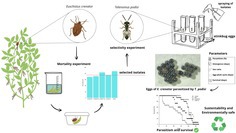

## 1 INTRODUCTION

Phytophagous stink bugs of the genus *Euschistus* Dallas (Hemiptera: Pentatomidae) are considered key pests in soybean cultivation [*Glycine max* (L.) Merrill], causing direct damage to the grains and compromising crop productivity.^1^, ^2^, ^3^ Among the species of this genus, *Euschistus crenator* (Fabricius, 1794) has been recorded in the northern regions of Brazil, in the states of Roraima and Pará.^4^ Owing to its morphological similarities with the brown soybean stink bug, *Euschistus heros* (Fabricius, 1798), this species is often misidentified in the field, which hinders the adoption of specific management strategies.^4^, ^5^, ^6^, ^7^, ^8^ This limitation is concerning because susceptibility to insecticides varies significantly among species and developmental stages.^9^, ^10^, ^11^, ^12^, ^13^, ^14^, ^15^ Considering that chemical control is still the main strategy employed in stink bug management,^2^, ^16^, ^17^, ^18^ identification errors may favor the evolution of resistant populations and negatively impact beneficial insects, especially when there are successive applications of broad‐spectrum insecticides.^8^, ^19^, ^20^, ^21^, ^22^, ^23^, ^24^, ^25^

Within the context of integrated pest management (IPM), biological control is a promising strategy for managing phytophagous stink bugs,^2^, ^26^, ^27^, ^28^ with particular emphasis on the use of the egg parasitoid *Telenomus podisi* Ashmead, 1893 (Hymenoptera: Scelionidae). This micro‐hymenopteran, measuring ≈1.0 mm in length, develops from egg to adult inside stink bug eggs^29^, ^30^, ^31^, ^32^ and exhibits high reproductive potential, allowing parasitism rates to exceed 80%, depending on the host species and environmental conditions.^26^, ^33^, ^34^, ^35^, ^36^ *T. podisi* is recognized for its ability to disrupt the life cycle of pests and its effectiveness in regulating pentatomid populations in agricultural systems.^30^, ^37^, ^38^, ^39^, ^40^, ^41^, ^42^, ^43^, ^44^, ^45^, ^46^ Considering that *E. crenator* exhibits biological parameters similar to those of *E. heros*,^4^ the preferential host of *T. podisi*,^4^, ^30^, ^47^ it is feasible to explore the potential of this parasitoid against *E. crenator*.

Phytophagous stink bug populations also can be regulated by biological control promoted by entomopathogens, with emphasis on entomopathogenic fungi (EPFs), which are characterized by their unique mode‐of‐action (MoA), which is characterized by their ability to actively penetrate the cuticle of insects.^48^, ^49^, ^50^, ^51^, ^52^, ^53^ Several fungal isolates have demonstrated high insecticidal potential for the biological control of stink bugs, representing a promising alternative to chemical insecticides.^48^, ^50^, ^54^, ^55^, ^56^, ^57^, ^58^ The most studied EPFs for controlling phytophagous stink bugs are *Metarhizium anisopliae* (Metschn.) Sorokin and *Beauveria bassiana* (Bals.‐Criv.) Vuill.^59^, ^60^, ^61^, ^62^ The latter shows high efficiency in controlling pentatomid species such as *E. heros*, *Piezodorus guildinii*, *Nezara viridula* and *Halyomorpha halys*, with mortality rates from 88% to 100%, depending on the isolate, concentration and exposure time.^48^, ^50^, ^55^, ^63^ However, for these agents to be effectively incorporated into IPM, they must be selective towards natural enemies, ensuring the conservation of ecosystem services.^64^, ^65^

The interaction between natural enemies is called intraguild and can be synergistic, additive or antagonistic, depending on the species involved, concentration and exposure time.^66^, ^67^, ^68^, ^69^ Therefore, the integration of different biological control agents requires careful attention, as negative interactions among system components can compromise management effectiveness.^70^, ^71^ In light of this, the present study was conducted to evaluate the insecticidal effect of fungal isolates on *E. crenator* nymphs and their selectivity to the egg parasitoid *T. podisi*.

## 2 MATERIALS AND METHODS

### 2.1 Obtaining and rearing of *Euschistus crenator*

Adults and eggs of *E. crenator* were obtained from the Insect Biology Laboratory at the ‘Luiz de Queiroz’ College of Agriculture, University of São Paulo (ESALQ/USP) in Piracicaba, São Paulo, Brazil. The colony was maintained in the laboratory under controlled conditions of temperature (27 ± 1 °C during the day and 26 ± 1 °C at night), relative humidity (RH, 65% ± 10%) and a photoperiod of 12 h:12 h, light:dark. Nymphs and adults were fed a natural diet consisting of green beans (*Phaseolus vulgaris* L.) and raw, shelled peanuts (*Arachis hypogaea* L.). Water was provided *ad libitum* via moistened cotton, which was replaced as needed.

### 2.2 Obtaining and rearing of *Telenomus podisi*

*Euschistus heros* eggs parasitized by *T. podisi* were provided by the Research Group on Integrated Pest Management in Agriculture (AGRIMIP) at the São Paulo State University ‘Júlio de Mesquita Filho’ (FCA/UNESP), Botucatu, São Paulo, Brazil. The newly emerged parasitoids were fed with droplets of honey and kept in a BOD‐type incubator under controlled conditions of temperature (27 ± 1 °C during the day and 26 ± 1 °C at night), relative humidity (65 ± 10%) and photoperiod (12 h:12 h, light:dark). For colony establishment, unparasitized eggs of *E. heros* from a laboratory colony were used. The eggs were affixed to strips of white cardboard (20 × 60 mm) using a 20% gum arabic solution.

### 2.3 Obtaining the fungal isolates and the bioinsecticide

The entomopathogenic fungal isolates LCMAP078 and LCMAP106 (*B. bassiana*), and LCMAP099 (*Penicillium bilaiae* Chalab.) were provided by the Entomopathogenic Microorganism Bank of the Laboratory of Microbial Control of Pest Arthropods (LCMAP) at the Faculty of Agricultural and Veterinary Sciences, São Paulo State University ‘Júlio de Mesquita Filho’ (FCAV/UNESP). The tested fungal isolates were deposited in the NCBI database (https://www.ncbi.nlm.nih.gov/nuccore) under accession nos PV656746 (LCMAP078), PV656748 (LCMAP099) and PV656747 (LCMAP106). The *B. bassiana* isolate (UFSM‐1) was supplied by the Soil Department of the Federal University of Santa Maria (UFSM).^50^ The isolates were stored in glycerol (10%) in an ultrafreezer at −80 °C. In addition to the isolates, the bioinsecticide Boveril Evo®, based on *B. bassiana* STRAIN PL63 (BOV) (Koppert Brasil, Paracicaba, Brazil), was used at the average dose recommended for *E. heros* (0.844 g a.i. L^−1^, equivalent to 1.7 × 10^6^ conidia mL^−1^). Suspensions were prepared by diluting fungal isolates in autoclaved deionized water containing Tween 80 (0.05%). Conidia counting was performed using a Neubauer chamber under a phase‐contrast microscope (Axio Lab.A1; Zeiss, Jena, Germany), and the concentration was standardized to 1 × 10^8^ conidia mL^−1^.

### 2.4 Mortality bioassay

For the tests, newly hatched *E. crenator* nymphs were fed and kept in Petri dishes (diameter 14 cm) until they reached the 2^nd^‐instar (± 5 days),^4^ an instar with high susceptibility to entomopathogen infection.^72^ A total of 400 2^nd^‐instar nymphs were used in the experiments.

Fungal suspensions were prepared by cultivating the isolates in Petri dishes (diameter 14 cm) containing potato dextrose agar (PDA) and pentabiotic (0.5 g L^−1^), kept in a BOD‐type incubator at 28 ± 2 °C for 10 days until conidiogenesis occurred. After this period, the cultures were scraped with a metal spatula, and the contents were diluted in autoclaved deionized water containing Tween 80 (0.05%).

In order to assess the bioactivity of the isolates, 100‐mL suspensions were prepared containing autoclaved distilled water, Tween 80 (0.05%), and the respective isolate at a concentration of 1 × 10^8^ conidia·mL^−1^, as well as the commercial strain PL63 (BOV) applied at the average dose recommended by the manufacturer (0.844 g a.i. L^−1^, equivalent to 1.7 × 10^6^ conidia·mL^−1^). Snap bean sections (± 5.0 cm) were immersed in the suspensions for 30 s, and after air‐drying, they were transferred to plastic containers lined with filter paper and sealed with voile fabric. The test arenas, containing the insects and treated pods, were maintained under controlled conditions of temperature (27 ± 1 °C during the day and 26 ± 1 °C at night), RH (65 ± 10%) and photoperiod (12 h:12 h, light:dark). The control treatment consisted of 2^nd^‐instar nymphs treated with autoclaved deionized water containing Tween 80 (0.05%). Snap beans were offered and replaced every 2 days for the insect feeding. Cumulative mortality was evaluated daily for 10 days, a timeframe within the mortality period of pentatomids caused by different *B. bassiana* isolates.^48^, ^62^, ^73^

### 2.5 Selectivity bioassay

Twenty‐five nonparasitized eggs of *E. crenator*, <48 h old, were used. The eggs were fixed onto strips of white cardboard (5.0 × 15 mm) using 20% gum arabic. For the selectivity tests, the fungal isolate LCMAP106, owing to its greater lethality against *E. crenator*, and UFSM‐01, owing to its efficiency in pest control and previous studies involving *E. heros*, another host species of *T. podisi*,^50^ were selected. Fungal suspensions were prepared at a concentration of 1 × 10^8^ conidia mL^−1^ in 100‐mL beakers containing autoclaved deionized water and Tween 80 (0.05%), in addition to the commercial strain PL63 (BOV) applied at the recommended average dose (0.844 g a.i. L^−1^, equivalent to 1.7 × 10^6^ conidia mL^−1^). The suspensions were applied inside glass tubes (25 × 85 mm) with the aid of a Potter Tower (Burkard, Hertfordshire, England) until runoff under constant air pressure (10.0 psi and 0.6895 bar).

After complete drying of the tubes in a laminar flow chamber (VECO, Campinas, Brazil), droplets of honey were added to the inner walls, and a mated *T. podisi* female was introduced. Fifteen mated females <48 h old were used per treatment, totaling 60 *T. podisi* females. The control consisted of autoclaved deionized water containing Tween 80 (0.05%) under the same conditions described above.

The females were kept in contact with the treated surface for 24 h, after which a card containing 25 unparasitized *E. crenator* eggs was added for parasitism. After 24 h, the parasitized eggs were removed and transferred to glass tubes without the presence of females. A second set of eggs was offered to assess the effects of the treatments after 72 h of exposure, according to the methodology described.^74^

The evaluated parameters were survival (days), parasitism rate (%), emergence rate (%), sex ratio and egg‐to‐adult cycle of the offspring (days), after 24 and 72 h of exposure of *T. podisi* females to the contaminated surface.

### 2.6 Data analysis

Bioassays were conducted using a completely randomized design (CRD). The data obtained were subjected to normality and homoscedasticity tests using the Shapiro–Wilk and Bartlett tests, respectively. Mortality data were previously transformed using √(*Y* + 0.5) or arcsine(√(*Y*/100)), depending on the nature of the dataset, to meet the assumptions of normality and homogeneity of variance before analysis using ANOVA (*P* ≤ 0.05). When significant, Tukey's multiple comparison test was performed at the 95% probability level. When the assumptions were not met, the nonparametric Kruskal–Wallis analysis was used, followed by multiple comparisons with the Mann–Whitney U‐test (*P* ≤ 0.05). The survival curve was constructed using daily survival data and based on the Kaplan–Meier methodology with the log‐rank test,^75^ and the lethal time for 50% mortality (LT_50_) was estimated using probit analysis. All statistical analyses were performed using SAS ondemand for academics software (SAS Institute, Cary, NC, USA), and graphs were produced using sigmaplot (SigmaPlot 12.3).

## 3 RESULTS

### 3.1 Mortality of *Euschistus crenator* by fungal isolates

**Figure 1:**
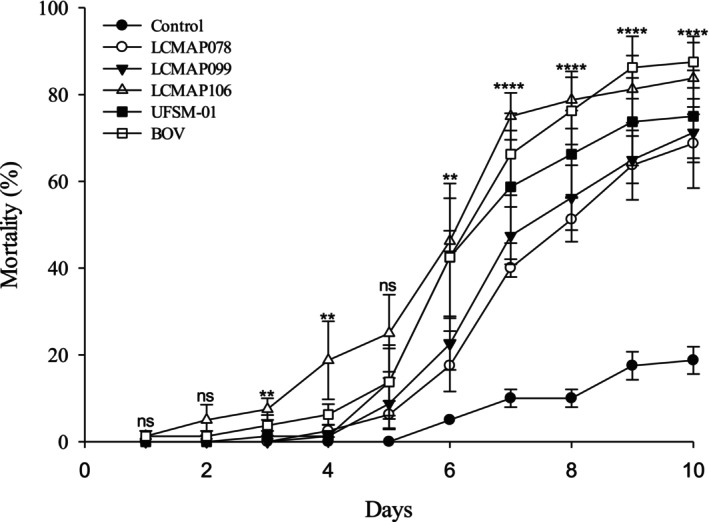
Cumulative mortality (%) of 2^nd^‐instar nymphs of *E. crenator* exposed to residues of different fungal isolates and the control (autoclaved deionized water) over 10 days of evaluation. Values are expressed as mean ± standard error. Significant differences between treatments and the control were determined using Kruskal–Wallis or ANOVA, followed by Mann–Whitney's U‐test or Tukey's test for multiple comparisons. Mortality data were transformed using √(*Y* + 0.5) or arcsine(√(*Y*/100)) when necessary. Asterisks indicate significant differences: **, *P* < 0.01;***, *P* < 0.001; and ****, *P* < 0.0001

The mortality rates of 2^nd^‐instar nymphs of *E. crenator* exposed to pods contaminated with fungal isolates of *B. bassiana* (LCMAP078, LCMAP106 and UFSM‐01), *P. bilaiae* (LCMAP099, and strain PL63 (BOV) are shown in Fig. 1. Three days postapplication (dpa), a significant difference was observed among the treatments (χ^2^ = 14.721; df = 5; *P* = 0.0116), with the LCMAP106 isolate showing the highest accumulated mortality (7.5 ± 2.5%), which differed from that of the control and the other isolates. At 4 dpa, LCMAP106 (18.75% ± 8.99%) maintained a significantly higher mortality, similar only to PL63 (BOV) (6.25% ± 2.39%), according to Tukey's test (*P* ≤ 0.01). At 6 dpa, LCMAP106 (46.25% ± 2.39%) showed significantly higher accumulated mortality than the other treatments and the control (5.0%) (*P* ≤ 0.01). The other treatments remained statistically similar to each other, with mortality ranging from 17.5% ± 5.95% (LCMAP078) to 42.5% ± 13.62% (BOV).

At 7 dpa, all treatments differed significantly from the control (*P* < 0.0001), and differences were observed among the isolates. The LCMAP106 isolate (75.0% ± 5.40%) showed the highest mortality rate, followed by BOV (66.25% ± 9.44%), UFSM‐01 (58.75% ± 12.97%), and LCMAP099 (47.5% ± 6.61%), whereas LCMAP078 (40.0% ± 2.04%) had the lowest mortality during this period. The control group had a mortality rate of 10.0% ± 2.04%. Between 8 and 10 dpa, all treatments showed significantly higher mortality than the control (*P* < 0.0001), ranging from 68.75% ± 10.28% (LCMAP078) to 83.75% ± 8.26% (LCMAP106) at 10 dpa. During the same period, the control showed a mortality of 18.75% ± 3.15%, whereas BOV showed 87.5% ± 5.95%.

The fungal isolates tested showed variations in the mean lethal time for 50% of the population (LT_50_). LCMAP78 exhibited the highest LT_50_ (8.224 ± 0.153 days), indicating a slower speed of action than the other treatments, whereas LCMAP99 (7.970 ± 0.213 days) and UFSM01 (7.326 ± 0.282 days) presented intermediate values. Finally, BOV (6.707 ± 0.181 days) and the *B. bassiana* isolate LCMAP106 (6.402 ± 0.208 days) showed the lowest LT_50_, demonstrating a faster effect on *E. crenator* nymphs than the other treatments (Table 1).

**Table 1:** Estimated LT₅₀ for 50% mortality of 2^nd^‐instar nymphs of *E. crenator* exposed to different fungal isolates

Trataments	LT_50_ ^a^ (days) ± SE^b^	95% CI (days)^c^
LCMAP078	8.224 ± 0.153	7.925–8.524
LCMAP099	7.970 ± 0.213	7.479–8.460
LCMAP106	6.402 ± 0.208	5.923–6.882
UFSM‐01	7.326 ± 0.282	6.676–7.975
BOV	6.707 ± 0.181	6.290–7.125

^a^ LT_50_ = Lethal time for the death of 50% of individuals.

^b^ SP = Standard error.

^c^ CI 95% – 95% confidence interval for TL50.

### 3.2 Selectivity of *Beauveria bassiana* isolates against *Telenomus podisi*

**Figure 2:**
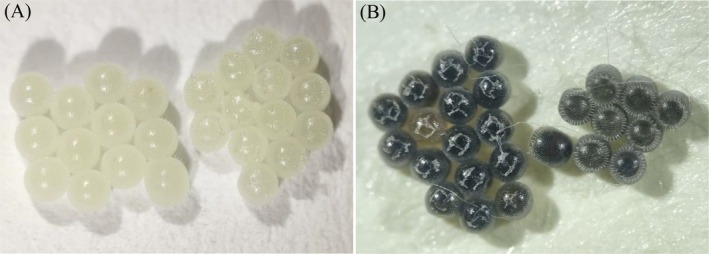
Morphology of *E. crenator* eggs: healthy (A) and parasitized (*T. podisi*) (B).

**Figure 3:**
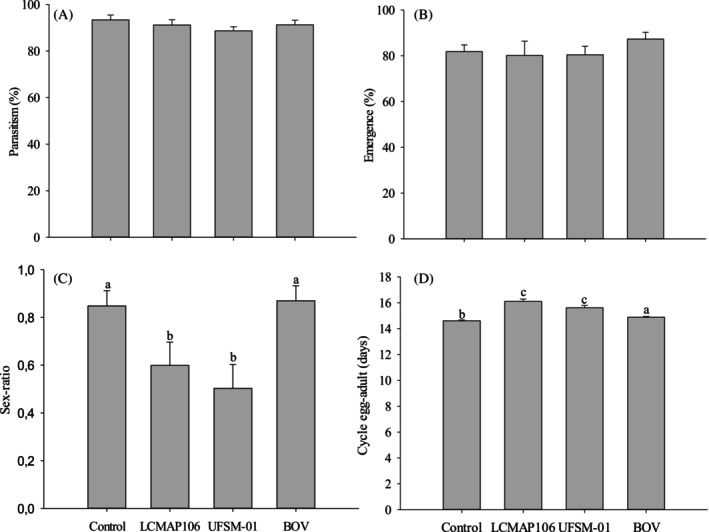
(A) Parasitism (%), (B) emergence (%), (C) sex ratio and (D) egg‐to‐adult cycle duration (days) of *T. podisi* offspring after 24 h of exposure of parasitoid females to different *B. bassiana* isolates (LCMAP106 and UFSM‐01) and to the commercial strain PL 63. Bars with the same letter do not differ significantly according to the nonparametric Kruskal–Wallis test (*P* ≤ 0.05). Error bars represent the standard errors of the mean.

**Figure 4:**
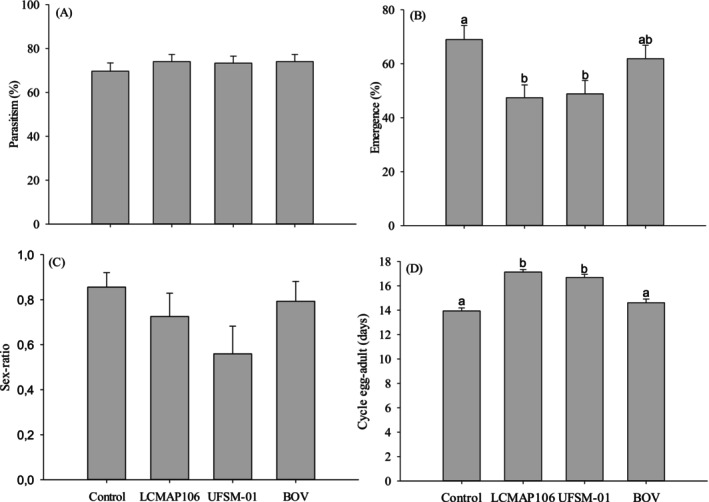
(A) Parasitism (%), (B) emergence (%), (C) sex ratio and (D) egg‐to‐adult cycle duration (days) of *T. podisi* offspring after 72 h of exposure to parasitoid females to different *B. bassiana* isolates (LCMAP106 and UFSM‐01) and the commercial strain PL 63. Bars with the same letter do not differ significantly according to Tukey's test (*P* ≤ 0.05). Error bars represent the standard errors of the mean.

The morphology of healthy and unparasitized *E. crenator* eggs is whitish‐yellow, whereas parasitized eggs display gray to black coloration (Fig. 2). The presence of fungal isolates did not alter the coloration of parasitized eggs, which remained similar to that of the control group. Contact between females and surfaces treated with *B. bassiana* isolates did not influence the average rate of egg parasitism by *T. podisi* (*P* > 0.05). The parasitism rates after 24 h were 93.37% ± 2.09% (control), 91.15% ± 2.28% (LCMAP106), 88.7% ± 1.76% (UFSM‐01), and 91.25% ± 2.02% (BOV) [Fig. 3(A)]. After 72 h, the average parasitism rates were 69.65% ± 3.77% (control), 74.04% ± 3.26% (LCMAP106), 73.32% ± 3.20% (UFSM‐01) and 74.04% ± 3.26% (BOV) [Fig. 4(A)].

The emergence rate of *T. podisi* offspring did not differ significantly between the treatments after 24 h, with rates ranging from 80.44% (LCMAP106) to 87.29% (BOV) (χ^2^ = 2.846; df = 3; *P* = 0.416), with values above 80.0% [Fig. 3(B)]. However, after 72 h, a significant reduction in emergence was observed for the treatments with LCMAP106 (47.42% ± 4.734%) and UFSM‐01 (48.84% ± 4.972%) compared to the control (68.97% ± 5.18%) and BOV (61.88% ± 4.97%), according to Tukey's test (*P* ≤ 0.01) [Fig. 4(B)]. The sex ratio of *T. podisi* offspring was significantly affected by the treatments after 24 h (χ^2^ = 23.612; df = 3; *P* < 0.0001), with the LCMAP106 (0.599% ± 0.098%) and UFSM‐01 (0.503% ± 0.10%) treatments resulting in a lower proportion of females compared to the control (0.848% ± 0.064%) and BOV (0.869% ± 0.063%) [Fig. 3(C)]. However, after 72 h, there were no significant differences in the sex ratio between the treatments (χ^2^ = 2.579; df = 3; *P* = 0.461), with all values ranging from 0.5589 ± 0.123 (UFSM‐01) to 0.856 ± 0.064 (Control) [Fig. 4(C)]. The developmental period from egg to adult was significantly affected by the treatments at both time intervals (*P* < 0.0001). The fungal isolates prolonged this period compared to the control group, whereas PL63 (BOV) led to an increased cycle only after 24 h [Figs 3(D) and 4(D)].

**Figure 5:**
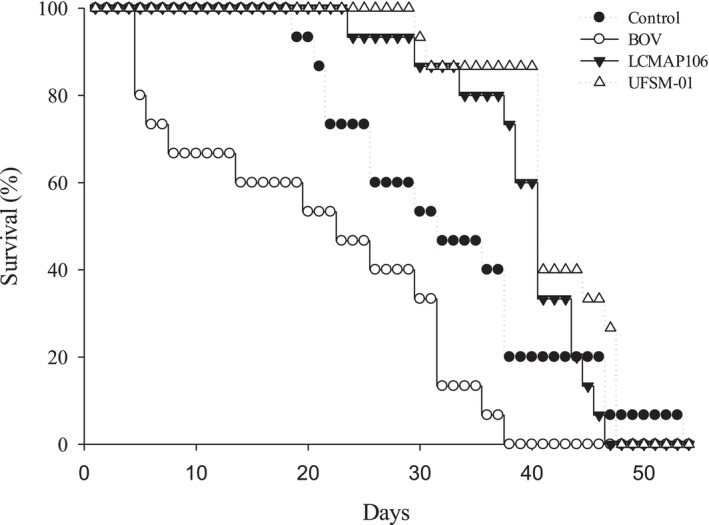
Survival curves of *T. podisi* females after exposure to different treatments: control, bioinsecticide based on *B. bassiana* (BOV), and fungal isolates LCMAP106 and UFSM‐01 (log‐rank test: χ^2^ = 0,6232; gl = 3; *P* = 0,8911).

Despite the visual tendency for higher early mortality in the BOV group, especially evident in the Kaplan–Meier curve (Fig. 5), statistical analysis using the log‐rank test did not indicate significant differences in the survival rates of *T. podisi* females exposed to the fungal isolates and the PL63 (BOV) strain of *B. bassiana* (log‐rank test: χ^2^ = 0.6232; df = 3; *P* = 0.8911). These results suggest that the bioinsecticide may affect the survival of *T. podisi* females, but this effect was not statistically detectable under the experimental conditions used. Survival analysis using the Kaplan–Meier method indicated that the LT_50_ ranged from 27.5 days for the commercial strain PL63 (BOV) to 32.0 days for the UFSM‐01 isolate, with 30.0 days for LCMAP106 and 28.0 days for the control group, with no significant differences between treatments (Table 2).

**Table 2:** Estimated survival of *T. podisi* females after exposure to different fungal isolates and the bioinsecticide based on *Beauveria bassiana*

Treataments	Mean±SE (days)	LT_50_ (days)^†^	95% CI (days)^‡^
Control	28.07 ± 2.00	28.0	22.0 – 35.0
BOV	26.88 ± 2.07	27.5	20.0 – 33.0
LCMAP106	28.66 ± 2.02	30.0	22.0 – 37.0
UFSM‐01	29.76 ± 1.97	32.0	24.0 – 39.0

^†^ LT_50_, lethal time for death of 50% of individuals.

^‡^ CI, 95% confidence interval for LT_50_.

## 4 DISCUSSION

This study demonstrated, for the first time, the potential of different EPFs in controlling *E. crenator*, a species for which there have been no records of the effectiveness of these biological agents. Although the action of EPFs on stink bugs has been widely reported, most related studies have sought to evaluate the insecticidal potential of these fungi by means of insect immersion or direct topical application on the pest.^48^, ^50^, ^57^, ^59^, ^63^, ^72^, ^76^, ^77^, ^78^, ^79^ The present study specifically aimed to evaluate the effect of surfaces treated with different fungal isolates, simulating conditions closer to those found in the field, in which spraying presents difficulties in reaching the target insect, and control depends largely on the pest's exposure to contaminated surfaces.^80^, ^81^, ^82^, ^83^, ^84^, ^85^ The results obtained broaden the scope of studies on the microbial control of stink bugs, including *E. crenator*, among the complex of target pests for these biological agents, which is particularly relevant in light of the growing demand for more sustainable and environmentally safe control methods.^86^, ^87^, ^88^, ^89^

A remarkable finding of this study was the mortality rate of 71.25% ± 5.91% in *E. crenator* nymphs caused by the *P. bilaiae* isolate LCMAP099. This finding is particularly significant, as there are no records of insecticidal activity of this species against soybean‐associated stink bugs. Unlike *B. bassiana*, which acts through active penetration of the insect cuticle, species of the genus *Penicillium* are widely recognized for producing toxic secondary metabolites with insecticidal properties.^90^, ^91^, ^92^ The insecticidal activity of *Penicillium*‐derived metabolites against insect pests suggests that mortality may be the result from ingestion or contact toxicity rather than cuticular infection.^93^, ^94^, ^95^ Therefore, the observed activity of *P. bilaiae* against *E. crenator* may be associated with the production of one or more bioactive metabolites, opening new perspectives for the biotechnological exploration of this genus as a source of novel natural insecticides.^96^, ^97^

In the present study, the control group exhibited performance within the expected survival parameters. Under laboratory rearing conditions, *E. crenator* nymphs fed on soybean pods may exhibit mortality rates close to 36% up to the adult stage.^4^ However, the UFSM‐01 isolate promoted a mortality rate of 75.0% ± 10.6% in *E. crenator*, a result comparable to that observed by other authors^50^ for *E. heros*, who reported 75% mortality by 9 dpa and 97% by 15 dpa of the same isolate. The use of *B. bassiana* in the management of *E. heros* has been explored, with mortality rates ranging from 70% to 90% under glasshouse conditions and reaching ≤97% in laboratory conditions, especially when concentrations between 1 × 10^8^ and 1 × 10^9^ conidia mL^−1^ are used.^50^, ^58^ These results corroborate the potential of *B. bassiana* as a biological control agent for different species within the stink bug complex.^56^, ^98^, ^99^

However, susceptibility to the same isolate can vary significantly between species because of physiological and behavioral differences.^100^, ^101^ For example, the emission of volatile compounds by stink bugs may have fungicidal or fungistatic effects, compromising the efficacy of the pathogen.^102^, ^103^ This intra‐ and interspecific variability reinforces the need for careful selection of isolates and prior characterization of the pathogen–host interaction, crucial steps for the development of more effective and selective formulations.^51^, ^104^, ^105^

The germination of EPF conidia occurs between 12 and 48 h after adhesion to the host's integument, provided that the temperature and humidity are adequate.^106^, ^107^, ^108^, ^109^ The destruction of the cuticle, which is necessary for insect death, usually occurs between 7 and 10 days after infection but may extend up to 21 days depending on the species.^110^, ^111^, ^112^ In the present study, a similar pattern was observed, with a progressive increase in mortality over time (Fig. 1). The more pronounced mortality between 6 and 10 days indicates that the action cycle of *B. bassiana* on *E. crenator* is compatible with that described for other stink bugs, such as *E. heros* and *Nezara viridula* (Linnaeus, 1758) (Hemiptera: Pentatomidae) and *Piezodorus guildinii* Westwood, 1837 (Hemiptera: Pentatomidae).^58^, ^103^, ^113^ Among the treatments, the commercial strain PL63 (BOV) was the most efficient, reaching 87.5% ± 5.95% mortality after 10 days, which reinforces the stability and potential of commercial formulations.^104^, ^114^

The selectivity of *B. bassiana* toward natural enemies including predators and parasitoids,^64^, ^115^, ^116^, ^117^ allows its integration with other biological control strategies, contributing to the sustainability of agricultural systems.^105^, ^118^, ^119^, ^120^ In this context, the egg parasitoid *T. podisi*, even after exposure to *B bassiana*‐based fungal isolates, maintained high parasitism rates, reaching >80% of *E. crenator* eggs within 24 h and over 70% after 72 h. The development of the parasitoids could be monitored by the change in coloration of the host eggs, which darkened as the adults emerged, as described.^40^ These results confirm previous studies^74^ that did not observe adverse effects on the parasitism of *E. heros* eggs by *T. podisi*, even after females were exposed to surfaces contaminated with *B. bassiana‐* and *M. anisopliae*‐based bioinsecticides. Although formulated products contain co‐formulants that may interfere with interactions with natural enemies,^121^, ^122^ in this study, the interaction was observed to be positive, and the parasitism of *E. crenator* eggs by *T. podisi* was not affected.

The emergence of *T. podisi* can be affected by several factors, including the age of the females, with a progressive decrease in the emergence of offspring observed as the females age.^123^ Fungi can reduce the quality of the host for parasitoid larvae, thus influencing the emergence rate of the offspring.^124^ This effect was observed in the reduction of offspring emergence percentages to 47.4% and 48.8%, caused by the LCMAP106 and UFSM‐01 isolates after 72 h. Nevertheless, parasitoids can secrete substances with fungistatic action in the host, preventing fungi from colonizing it and thus ensuring the emergence of their offspring.^67^

The sex ratio of egg parasitoids is influenced by factors such as the age of the female and the quality of the host egg.^30^, ^125^ In the present study, the fungal isolates altered the sex ratio, which approached 0.5 after 24 h of exposure. Reductions in the sex ratio can negatively impact stink bug control, as only females are parasitic.^126^ The development time from egg to adult emergence for *T. podisi* lasts an average of 10–14 days at temperatures of 25–32 °C.^127^ Spraying *B. bassiana* isolates onto *T. podisi* and *Telenomus remus* Nixon, 1937 (Hymenoptera: Scelionidae) did not alter the egg‐to‐adult period in previous studies.^74^, ^128^ Although some isolates may reduce or prolong this period,^129^, ^130^, ^131^ small changes are unlikely to affect the population maintenance of natural enemies in the field.^132^

No significant differences were observed in the survival time of adult *T. podisi*, regardless of whether they were exposed to fungal isolates. Under laboratory conditions, the longevity of adults of this species can range from 3 to 54 days, depending on factors such as nutrition and the availability of host eggs.^40^, ^133^ This variation can be influenced by a variety of factors, including the direct effects of nonselective entomopathogens, as well as environmental conditions such as temperature, humidity and host quality.^29^, ^42^, ^130^, ^134^, ^135^ However, the survival values obtained in this study indicate that the tested fungal isolates were selective for the parasitoids, even under conditions of high exposure, such as those applied in laboratory tests.^136^, ^137^, ^138^ These results indicate the potential for integration between EPFs and egg parasitoids, promoting more sustainable and ecologically sound pest management.^49^, ^139^, ^140^

## 5 CONCLUSION

The results obtained demonstrate the potential of *B. bassiana* isolates in the biocontrol of *E. crenator*, with special emphasis on the performance of LCMAP106 and UFSM‐01 isolates. Regarding the selectivity of the isolates toward the egg parasitoid *T. podisi*, despite the reduction in offspring emergence rate and changes in the sex ratio of the parasitoids, the impact on the performance of *T. podisi* in terms of parasitism was minimal. This indicates that the association between egg parasitoids and the use of entomopathogenic fungi is promising for the management of *E. crenator*.

## CONFLICT OF INTEREST

The authors declare that they have no competing financial interests or personal relationships that could have influenced the work reported in this study.

## AUTHOR CONTRIBUTIONS

Paulo Henrique Martins da Silva: Contributed to the conception, design, acquisition and interpretation of data, was involved in drafting the manuscript and agreed to be responsible for aspects related to the work, such as its accuracy and integrity. Gustavo Andrade Carneiro: Contributed to the conception, design, acquisition and interpretation of data, and critical review of the manuscript. Ricardo Antônio Polanczyk: Contributed to the conception and design of the work, and was involved in the critical review of the manuscript for important intellectual content and final approval of the version to be published.

## Data Availability

The data that support the findings of this study are available from the corresponding author upon reasonable request.
